# Generation of acoustic-Brownian noise in nuclear magnetic resonance under non-equilibrium thermal fluctuations

**DOI:** 10.1038/s41598-020-77206-8

**Published:** 2020-12-08

**Authors:** Dhiraj Sinha

**Affiliations:** 1 Department of Electrical Engineering and Computer Science, Massachusetts Institute of Technology, 77, Massachusetts Avenue, Cambridge, MA 02139 USA; 2 Reimagining Higher Education Foundation, 302, Gopal Heights, Netaji Subash Place, New Delhi, 110034 India

**Keywords:** Biomedical engineering, Chemical physics

## Abstract

We present an analytical study on generation of acoustic-Brownian noise in nuclear magnetic resonance (NMR) induced as a result of thermal fluctuations of the magnetic moments under non-equilibrium thermal interactions which has not been explored independent of Nyquist–Johnson noise until now. The mechanism of physical coupling between non-equilibrium thermal fluctuations and magnetic moments is illustrated using Lighthill’s formulation on suspension dynamics. We discover that unlike Nyquist–Johnson noise which has a uniform spectral density across a range of frequencies, the spectral dependence of acoustic-Brownian noise decreases with an increase in frequency and resembles Brownian noise associated with a particle in a potential well. The results have applications in the field of image enhancement algorithm as well as noise reduction instrumentation in NMR systems.

## Introduction

Enhanced signal to noise ratio is one of the most vital aspects of high resolution nuclear magnetic resonance (NMR)^1^. It is closely associated with resolution of images in magnetic resonance imaging which is critical to detection of vital physiological conditions like cancer^2^, sclerosis^3,4^ neural injury^5^ and Alzheimer’s disease^6^. Presence of noise reduces the quality of images leading to erroneous interpretation of vital data related to the target sample which has critical healthcare implications. In order to reduce the overall effect of noise, signal enhancement in NMR is achieved using experimental techniques like polarization transfer^7^, nuclear Overhauser effect^8^, magic-angle sample spinning, multiple-pulse and proton-enhanced nuclear induction^9^. It also involves the use of advanced signal processing tools comprising Fourier and Radon transforms^10,11^.

Besides research on signal enhancement techniques in NMR, some studies have been done on understanding the impact of thermal fluctuations on magnetization and its relaxation. The impact of thermal fluctuations on magnetization curve in single domain materials was reported by Brown^12^. Carr and Purcell reported a detailed study of the role of diffusion on free precession and relaxation time in NMR^13^. The impact of spatial drift of nuclei under thermal fluctuations and its role in modulating the precession frequency and NMR spectra was reported by Wessner^14^. A related work considered fluctuation of the static magnetic field as a consequence of interaction with a thermal source and its role on spin interaction^15^.

Existing literature on calculation of signal to noise ratio in NMR rely on statistical analysis of mean value of signal and spectral distribution of noise derived from the final image^16^. Signals have a regular pattern which are enhanced under multiple averaging whereas noise signals cancel out due to random pattern^17^. These models of noise in physical systems are based on the work of Nyquist^18^ and Johnson^19^ which predict a uniform spectral power density of noise. Such noise models are also used in analyzing noise generated in the radio frequency coils of NMR^20^.

In recent years, research on MRI has moved towards the use of ultrahigh magnetic fields (7T) operating at 298.06 MHz, with the objective of increasing image resolutions^21,22^. At the same time, there has been a significant effort towards development of portable MR systems operating at fields below 10 MHz, with relatively low resolution images for point of care diagnostics^23,24^. Thus, the overall bandwidth of operation of NMR instrumentation is increasing, which calls for novel theoretical and empirical models on noise. In that context, development of novel develop analytical models on thermal fluctuations on a magnetized material sample can offer new insights in signal to noise ratio enhancement over a specific frequency band.

In a related technique, called nuclear acoustic resonance (NAR), the transition between energy levels associated with the orientation of nuclear spins polarized under external magnetic and electric fields is induced under acoustic energy transfer. In NAR, the coupling between, acoustic waves and nuclei is achieved under modulation of magnetic or electrical multipole moments^25^. The nuclear spins interact with external phonons under spin-lattice relaxation mechanism under thermal effects leading to de-excitation of spins^25^. At room temperature, the effect is trivial, but it can be dominant if acoustic energy is introduced into the system at significant power levels, where, the nuclei subjected to an external magnetic field, absorb acoustic waves. The phenomenon is mainly observed in metals, for example, it was initially observed in the context of aluminum^26^. In metals, the dominant mechanism of coupling is time varying electric field gradient generated by ultrasonic wave and dynamic dipolar interaction, where acoustic waves induce microscopic currents and time varying radio frequency magnetic fields, which interact with magnetic dipole moments^27^. The magnetic spin of nuclei, polarized under an external static field, can absorb acoustic waves under spin-lattice interaction. Nuclear acoustic resonance is observed in an indirect manner, as a change in amplitude of acoustic waves under absorption^28^. Acoustic attenuation differences in NAR is of the order of 1/1000, which is below the detection range of nuclei spin systems. Detection of NAR in liquids is impossible, as thermal fluctuations influence the field gradients. Despite intense work in the field of NAR, the fundamental aspect of fluctuation of magnetization as a consequence of acoustic wave propagation fields generated by acoustic sources under thermal interactions and its role in generation of noise and the way fluctuation dissipation theorem is applied in an NMR system remains unexplored, which is the central goal of the current work.

## The dynamics of nuclear spin in a magnetic field

In order to study the effect of thermal fluctuations and acoustic fields on magnetic spin, we initially consider its dynamics under static and time varying magnetic field. The dynamics of magnetic spin of a sample comprising of atoms, molecules or nuclei with a magnetization $\varvec{M}$ placed in a static magnetic field $\varvec{B}_0$, is given by^29^,

1 $$ \frac{d\varvec{M}}{dt}=\gamma \varvec{M} \times \varvec{B}_0 $$

where, *t* is time and $\gamma$ is the gyromagnetic ratio. Each of the individual samples with a magnetic dipole moment $\varvec{m}_m$ experience a torque, $\varvec{T}_q= \varvec{m} \times \varvec{B}_0$. The magnetic moment for Hydrogen ion is $1.41 \times 10^{-26}\,\hbox {J/T}$ and the value of its $\gamma$ is $42.58\,\hbox {MHz/T}$ . Considering a static magnetic field of 1 T, the torque comes out to be $1.41 \times 10^{-26}\,\hbox {Nm}$^21^. The magnetization in a Cartesian coordinate system can be expressed as, $\varvec{M}=M_x \hat{\varvec{x}}+M_y \hat{\varvec{y}}+M_z \hat{\varvec{z}}$, where $M_x$, $M_y$ and $M_z$ are the components of magnetization along $\hat{\varvec{x}}$, $\hat{\varvec{y}}$ and $\hat{\varvec{z}}$ directions. Similarly, the magnetic flux density with its components $\varvec{B}_{0x}$, $\varvec{B}_{0y}$, and $\varvec{B}_{0z}$ along the three coordinate vectors is written as, $\varvec{B}=B_{0x}\hat{\varvec{x}}+B_{0y} \hat{\varvec{y}}+B_{0z} \hat{\varvec{z}}$ . As the *z* component of magnetization does not vary, $d\varvec{M}_z/dt=0$. The relationship between static magnetic field and precession frequency is, $\omega _0=\gamma \varvec{B}_{0}$. While using these relations in Eq. 1, we get,

2 $$ \frac{d\varvec{M}}{dt}=\gamma B_{0z}[M_y\hat{\varvec{x}}-M_x\hat{\varvec{y}}]=\omega _0 \varvec{M} $$

Interaction between a given system and an external thermal source can lead to fluctuations in the magnetization^15^. However, the exact mechanism of coupling between a thermodynamic field and magnetic forces of the sample and its overall impact on the physical dynamics need to incorporate the effect of acoustic wave propagation and related effects. It is well established that thermal fluctuations lead to generation of an acoustic wave in a given medium^30^. In solids, there is a large body of knowledge on spin–phonon interaction^31,32^ which leads to interesting physical effects like acoustic paramagnetic resonance in transition metals^33,34^. Acoustic waves influence the dynamics of charged particles and long range electron transfer under the impact of surface acoustic waves have been reported^35^. However, the effect of acoustic waves and related sources in noise generation in nuclear magnetic resonance spectroscopy has not been reported in current literature. The conductivity of the sample is considered to be the major source of electrical noise in NMR systems.

The nuclei of ionized Hydrogen atoms in a fluid medium like water, move freely while carrying a positive charge. The overall dynamics at a given equilibrium can be described by a root mean square velocity. A critical question is whether thermal fluctuation induced by acoustic waves can influence the spatial orientation of such a nuclei. It is well known that the magnetic field of atoms in a molecule are screened by the presence of electron cloud^36^, which causes a shift in resonance frequency of the output signal in magnetic resonance spectroscopy. The external magnetic field is partially shielded by the electron cloud at the position of the nucleus, which results in a shift in resonant frequency. The magnetic field generated and the changes in magnetization are also influenced by the viscosity of the medium. Experiments have proved that the relaxation of magnetic spin is affected by the viscosity of the medium, for example, when the viscosity of the medium is high, both the longitudinal (or spin-lattice) relaxation time, $T_1$, which is the decay constant for the recovery of the *z* component of the magnetization, $M_z$, towards its thermal equilibrium value as well as the transverse (or spin-spin) relaxation time $T_2$, which is the decay constant for the component of $\varvec{M}$ perpendicular to $B_0$, increase in magnitude^37^.

**Figure 1:**
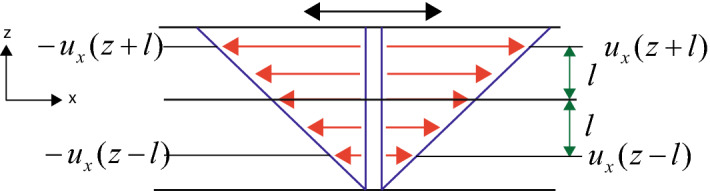
Transfer of momentum across a plane as a consequence of motion of molecules. The upper plane is periodically moved along the $$+ x $$ and $$ -x $$ direction. The velocity components are dependent on the coordinate positions.

In order to examine the overall impact of acoustic waves on the dynamics of molecules in a fluid, we consider a fluid enclosed between two parallel plates as shown in Fig. 1, where a pressure $$ P_z $$ is exerted on the upper plane in the *z* direction. The pressure along the *x* direction$$ P_{zx} $$ is zero, if the fluid is in equilibrium^39^. At a given time, $$ t=0 $$, we start moving the upper plate along the $$ +x $$ and $$ -x $$ direction in an oscillatory motion. At a given instance of time *t*, when the upper plate is moved in the $$ +x $$, direction, the velocity of fluid at a given value of *z* is $$u_x(z) $$. If there are *n* molecules per unit volume, we can assume that *n*/3 have a velocity component in the *z* direction. Half of these molecules i.e. *n*/6 molecules per unit volume have a mean velocity $$ {\bar{v}}\ $$ in the $$ +z $$ direction and the remaining half *n*/6 have a mean velocity $$ {\bar{v}}\ $$ component in the $$ -z $$ direction. Hence, on an average, the number of molecules crossing a unit area of the plane $$ z =$$ constant, from below, per unit time is $$ n {\bar{v}}/6 $$. Such molecules had their last collision at a distance *L*, where *L* is the mean free path of collision. The molecules at the given position, had a mean *x* component of velocity $$ u_x(z-L) $$. It implies that the mean *x* component of momentum transfer per unit time per unit area through the plane $$ z=0 $$ along $$ +z $$ direction is $$ n {\bar{v}} mu_x(z-L)/6 $$. The corresponding value for momentum transfer through the plane along the downward direction is $$ n {\bar{v}} mu_x(z+L)/6 $$. The net transfer of momentum along the upward direction is,

3 $$ P_{zx}=\frac{1}{6} n {\bar{v}} m[u_x(z-L)-u_x(z+L)] $$

Using Taylor expansion of the velocity, we can write, $u_x(z+L)=u_x(z)+(\partial u_x/\partial z) L$ and $u_x(z-L)=u_x(z)-(\partial u_x/\partial z) L$. Thus, we get the following expression,

4 $$ P_{zx}=-\eta \frac{\partial u_x}{\partial z} $$

where, $\eta = n {\bar{v}} mL/3$. The molecules are supposed to have a finite spherical shape and the relationship between mean free path and scattering cross sectional radius, $\sigma _0$, is $L=1/(\sqrt{2}n \sigma _0)$^39^. It leads to,

5 $$ P_{zx}=-\frac{m{\bar{v}}}{3\sqrt{2} \sigma _0} \frac{\partial u_x}{\partial z} $$

The average velocity is dependent on temperature, *T* and is expressed as, ${\bar{v}}=\sqrt{8k_{\mathrm {B}}T/\pi m}$.

**Figure 2:**
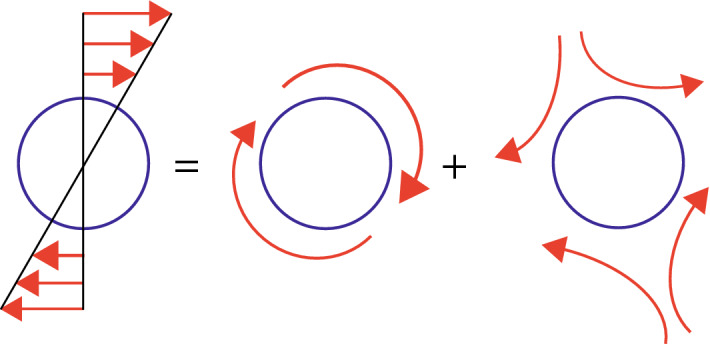
A sphere in shear can be decomposed into a sphere in rotation and a sphere in stress.

The velocity $u_x$ is dependent on time, hence, under a periodic motion of the upper plane of Fig. 1, an acoustic wave is generated in the fluid. A time and space dependent velocity field can be associated with the acoustic wave propagating in the medium which can influence rigid bodies in its path of propagation. According to Lighthill’s analysis, the velocity field associated with a rigid body of finite size, under a specific value of shear stress can be decomposed into a sum of uniform translation and a linearly varying field, which is represented mathematically as^40,41^,

6 $$ \varvec{u}(x)=\varvec{U}^\infty +\varvec{\Omega }^\infty \cdot \varvec{x} + \varvec{E}^\infty \cdot \varvec{x} $$

Here, $\varvec{U}^\infty$ is linear velocity $\varvec{\Omega }^\infty$ is an asymmetric rate of rotation tensor defined as,

7 $$ \Omega ^\infty _{ij}=\frac{1}{2}\left[ \frac{\partial u_i}{\partial x_j} -\frac{\partial u_j}{\partial x_i}\right] $$

where, *i* and *j* are indices which take on values, 1, 2 or 3 in three dimensions, $u_i$ and $u_j$ are velocity components along $x_i$ and $x_j$ directions and $\varvec{E}^\infty$ is symmetric rate of strain tensor which can be written as,

8 $$ E^\infty _{ij}=\frac{1}{2}\left[ \frac{\partial u_i}{\partial x_j} +\frac{\partial u_j}{\partial x_i}\right] $$

The possibility that viscous forces can influence the rotation of a nucleus under fluctuations generated by acoustic field can be analysed by incorporating the molecular dynamics using kinetic theory of gases. The acoustic waves induced by thermal fluctuations propagating in a medium can modulate viscosity through a change in velocity of the molecules leading to an additional drag force at the molecular level, which can, in principle, propagate to the level of individual nucleus of molecules.

The shear force on a molecule can be written as a sum of rotation and strain (Fig. 2),

9 $$ \varvec{\nabla }u=\varvec{\Omega }^\infty + \varvec{E}^\infty $$

The rate of rotation tensor is the rotation vector or the vorticity, defined as,

10 $$ \varvec{\omega }_i^\infty =-\frac{1}{2} \varvec{\epsilon }_{ijk} \varvec{\Omega }_{jk}^\infty =\frac{1}{2}\frac{\varvec{\nabla }\times \varvec{u}^\infty }{2}=\frac{1}{2} \varvec{{{\tilde{\omega }}}_i} $$

Here, $\epsilon _{ijk}$ is the Levi Civita tensor with indices, *i*, *j*, *k*, which take on values from 1 to 3 in three dimensions. Thus, the fluid can exert three different kinds of effects: rotation, translation and straining. The torque $\varvec{T}$ on a sphere in a fluid is expressed as^40,41^,

11 $$ \varvec{T}(t)=8 \pi \mu a^3 \left[ \frac{1}{2}\varvec{\nabla }\times \varvec{u}'-(\varvec{\Omega }- \varvec{\Omega }^\infty )\right] $$

**Figure 3:**
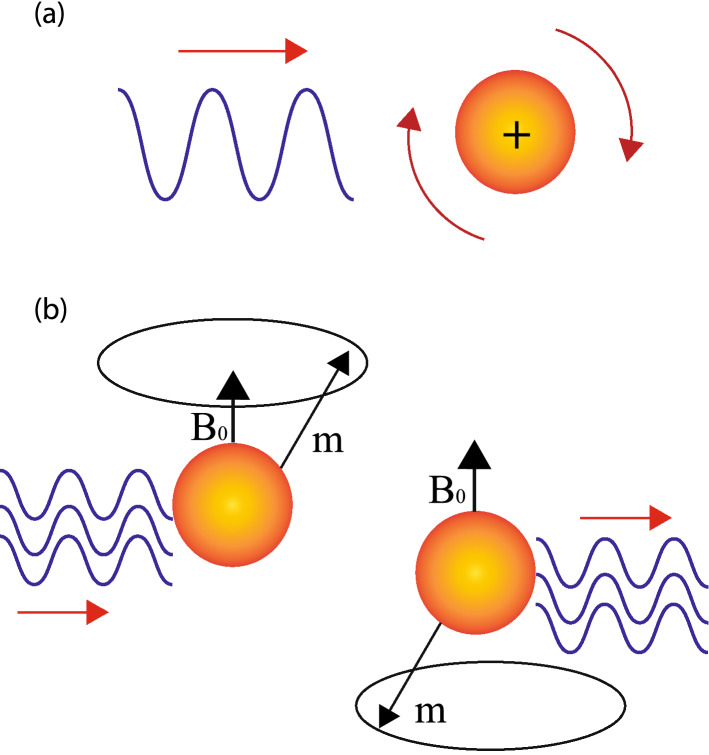
The rotational impact of acoustic waves on Hydrogen nucleus. (**a**) A Hydrogen nucleus with a positive charge can oscillate in a fluid medium under acoustic excitation. The viscous forces associated with oscillations can induce rotational motion. (**b**) The magnetic spin of Hydrogen nucleus under external magnetic field can get coupled to acoustic waves through shear forces due to viscosity of the media.

Here, $\varvec{u}'$ is the disturbance velocity induced by other spheres in suspension which is evaluated at the centre of sphere, $\varvec{\Omega }$ is the angular velocity of the sphere, $\varvec{\Omega }^\infty$ is the angular velocity of the background flow evaluated at the centre of the sphere. Thus, the impact of an acoustic wave on molecules in fluid is to rotate it and a Hydrogen nuclei in fluid can undergo rotation under acoustic excitation (Fig. 3a). The additional torque on the nuclei as a consequence of acoustic wave can influence radiation characteristics of a magnetized nuclei in a static field (Fig. 3b).

Considering, $\varvec{u}'=0$ and substituting $\varvec{\Omega }- \varvec{\Omega }^\infty =10^6\,\hbox {Hz}$, and assuming the dynamic viscosity of water as $8.90 \times 10^{-4}$ Pas, the radius of Hydrogen nucleus as 1.2 pm, the net torque comes out to be $3.87\times 10^{-25}\,\hbox {Nm}$ under the effect of an acoustic wave having a net angular frequency of 1 MHz. It is stronger in comparison to the torque generated by a magnetic field at 1 T calculated earlier which is $1.41\times 10^{-26}\,\hbox {Nm}$. Such large values of torque would be confined only to ultra-small MRI systems. For example in a tube of volume $1\,\hbox {mm}^3$, comprising water, the velocity of sound is 1450 m/s, the first resonant acoustic mode has a frequency of $1.45\,\hbox {MHz}$. However, for a typical square volume of $1000\,\hbox {cm}^3$, the resonant frequency is $1.45\,\hbox {kHz}$ and the noise magnetization would be extremely low. Hence, the current analysis could be of interest for noise calculations in miniaturized MRI systems for small volume diagnosis which are an active area of research. The calculations have been done in the context of a single nucleus of Hydrogen, however, it is worth pointing that and such calculations represent a simplified perspective of a complex system whose physical behavior is governed by the principles of statistical mechanics. Viscous effects are also the statistical outcome of the interaction of a large number of molecules.

The Langevin equation for a sample under the effect of static magnetic field and acoustic fluctuations can be written as,

12 $$ \varvec{T}=\frac{1}{\gamma }\frac{d\varvec{M}}{dt}=\varvec{M} \times \varvec{B}- \eta \varvec{\omega }+ \varvec{\xi }(t) $$

Here, $\eta$ is the damping coefficient. The fluctuating torque $\varvec{\xi }(t)$, which is defined as^38^,

13 $$ \langle \varvec{\xi }(t)\varvec{\xi }(t')\rangle =2\eta k_{\mathrm {B}} T \delta (t-t') $$

Multiplying both sides of Eq. 10 by $\gamma$ and separating the *x* and *y* components we get,

14 $$ \frac{d M_x}{dt}=\omega M_y- \eta \gamma \omega + \gamma {\xi }(t) $$

15 $$ \frac{d M_y}{dt}=\omega M_x- \eta \gamma \omega + \gamma {\xi }(t) $$

**Figure 4:**
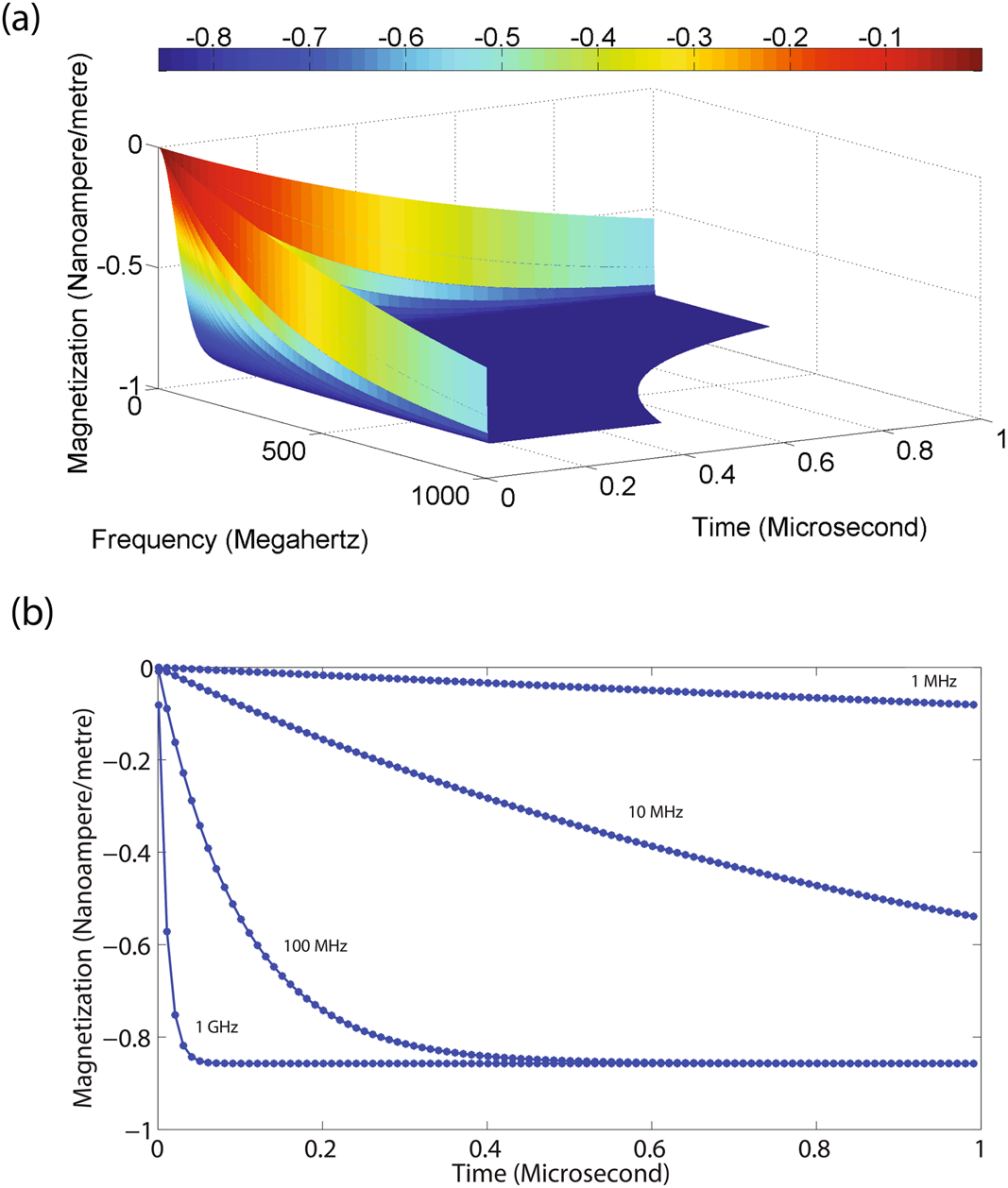
Magnetization noise in NMR (**a**) The noise level (in units of nA/m) shows an exponential decrease with an increase in frequency and time eventually reaching a stationary value. The frequency was varied from $1\,\hbox {MHz}$ to $1\,\hbox {GHz}$ and time was varied from 0 s to $1\,\upmu \hbox {s}$. (**b**) The time dependence of noise at some select precession frequencies viz. $1\,\hbox {MHz}$, $10\,\hbox {MHz}$, $100\,\hbox {MHz}$ and $1\,\hbox {GHz}$ also shows an exponential decrease with time. The total value of noise increases as the precession frequency increases.

The solutions of Eqs. 12 and 13 can be written as,

16 $$ M_x= \frac{e^{-\omega t}}{2\omega }[-2\gamma \xi +2e^{\omega t}\gamma \xi M_{x0}\omega + M_{x0}\omega e^{2\omega t}+ M_{y0}\omega - M_{y0}\omega e^{2\omega t}+2\eta \gamma \omega -2e^{\omega t}\gamma \eta \omega ] $$

17 $$ M_y= \frac{e^{-\omega t}}{2\omega }[2\gamma \xi -2e^{\omega t}\gamma \xi + M_{x0}\omega + M_{x0}\omega e^{2\omega t}- M_{y0}\omega - M_{y0}\omega e^{2\omega t}+2\eta \gamma \omega +2e^{\omega t}\gamma \eta \omega ] $$

We can assume the magnetization values $M_{x0}=M_{y0}=0$ at a given equilibrium. At a temperature, $T=300\,\hbox {K}$ and damping coefficient of 0.01, a graphical illustration of Eq. 16, is shown in Fig. 4a. The noise level is represented in units of nA/m which decreases exponentially with an increase in frequency from $1\,\hbox {MHz}$ to $1\,\hbox {GHz}$ as well as time which varies from $0\,\hbox {s}$ to $1\, \upmu \hbox {s}$. It finally reaches a stationary value. The slices of noise at some select precession frequencies viz. $1\,\hbox {MHz}$, $10\,\hbox {MHz}$, $100\,\hbox {MHz}$ and $1\,\hbox {GHz}$ are shown in Fig. 4b, which shows a drop in the level of noise with an increase in frequency. The level of noise also drops exponentially before saturating to a final value with an increase in time. This is because the magnetization decreases exponentially with an increase in frequency as well as time. The magnetization noise generated as a consequence of thermal fluctuations is different from white noise which has a uniform spectral response.

The majority of molecules in water are found in the molecular configuration of $\hbox {H}_2\hbox {O}$, which is highly stable. A tiny percentage of molecules are present as hydrogen and hydroxide ions. Hydrogen ions have a high value of charge density, and they hydrate in a solution forming a hydroxonium ion ($\hbox {H}_3\hbox {O}^+$)^36^. In a typical sample, with a pH of 7, the total hydrogen ion concentration is $10^{-7}$ moles per litre and the corresponding number of ions is $6.023 \times 10^{17}$ per litre. As the magnetic moment of proton is $14.106 \times 10^{-27}\,\hbox {A m}^2$, the total magnetization comes out to be $14.106 \times 10^{-10}\,\hbox {A m}^2$ or $1.4\,\hbox {nA/m}$ at room temperature conditions. The thermal fluctuation induced acoustic magnetization is in the range of a fraction nA/m (Fig. 4), which is lower than magnetization of hydrogen in water by a factor of 10 and decays in an exponential manner.

## Mode enhancement under feedback

**Figure 5:**
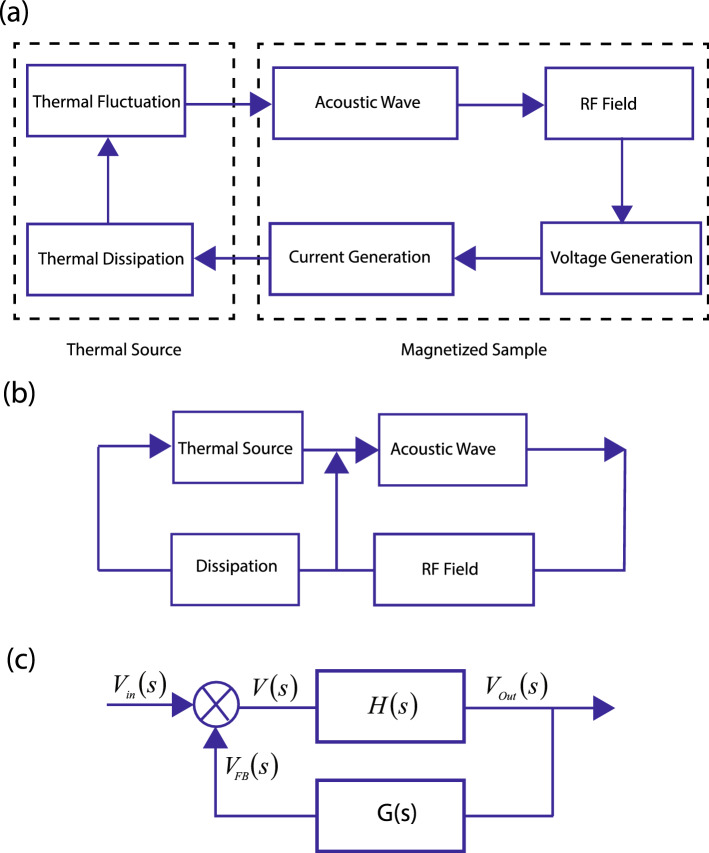
Energy enhancement under feedback. (**a**) Thermodynamic fluctuations from a thermal source get coupled to a fluid system leading to generation of acoustic wave which generates an RF field. The voltage and current is eventually dissipated as heat which is fed to the thermal source. (**b**) The energy can oscillate between acoustic and RF field under interation with a thermal source (**c**) At the start of the measurement process, at time $$ t=0,$$ the noise voltage in the frequency domain, $$V(s)=V_{{\rm in}}(s)$$. At the output, the voltage $$V_{{\rm out}}(s)$$ appears under an interaction between the input voltage and the system’s transfer function. The net voltage fed at the input is expressed as $$V_{{\rm FB}}(s)$$ which is algebraically added to the input voltage $$V_{{\rm in}}(s)$$, which is associated with thermal noise.

The signal obtained under acoustic excitation of magnetized sample can be enhanced under interaction with the existing modes in a system. A given material of finite elasticity generates acoustic modes under thermal interaction with a thermal source. According to the fluctuation dissipation theorem, the acoustic spectrum generated under thermal fluctuations results in power dissipation which can be written as, $P_N=k_{\mathrm {B}}T\Delta f$^38^ and $\Delta f$ is the bandwidth of frequency. For a homogenous system of infinite dimensions, the spectral power density of the acoustic noise signal is independent of frequencies. However, in a magnetized sample under interaction with a heat bath, thermal fluctuations lead to transient acoustic waves, which modulates the orientation of magnetic moments leading to localized generation of radio frequency waves which induces voltage and current which are injected into the thermal source and dissipated, thus increasing the level of thermal fluctuations which is again fed back into the magnetized material as illustrated in Fig. 5a. A part of the RF energy directly goes on to generate acoustic waves at the given frequencies (Fig. 5b). Thus, the system can have distinct acoustic and electromagnetic modes which get enhanced under fluctuation and dissipation during interaction with the thermal source.

The magnetized material comprising of a number of modes can be expressed using a transfer function *H*(*s*) where the feedback of energy from the output to its input is expressed using another transfer function *G*(*s*) as schematically illustrated in Fig. 5c. The input voltage to the magnetic material expressed as, voltage *V*(*s*) represents the frequency domain representation of noise voltage. At the onset of the measurement process, $V(s)=V_{{\rm in}}(s)$, which results in an output noise voltage expressed as, $V_{{\rm out}}(s)$ due to an interaction between the input voltage and the system’s transfer function. The output voltage is transferred to the input terminal under interaction with the transfer function of the feedback part expressed as *G*(*s*) . Thus, the net feedback voltage is $V_{{\rm FB}}(s)$ which is algebraically added to signal $V_{{\rm in}}(s)$.

**Figure 6:**
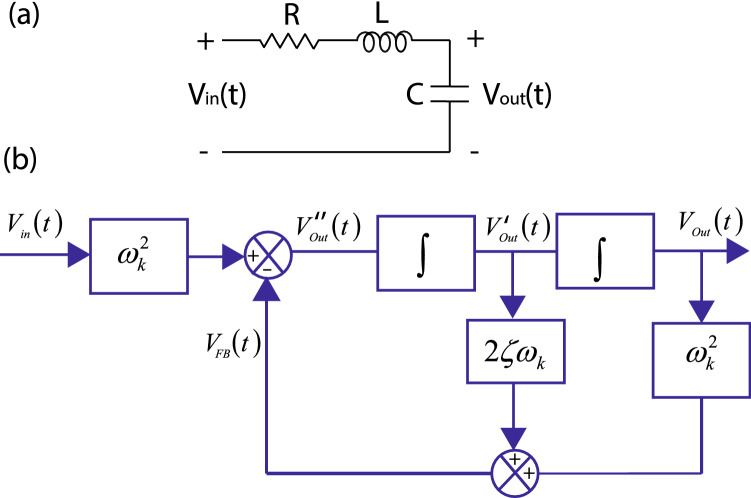
Circuit based model of feedback. (**a**) A resistor *R*, capacitor, *C* and an inductor *L* in series represents the circuit equivalent model of a given specimen subjected to an input voltage radio frequency voltage $V_{in}$. (**b**) A block diagram based representation of Eq. 18 is shown here. $V''_{Out}(s)$ and $V'_{Out}(s)$ denote the second and the first derivative of output voltage. $V_{Out}$ and $V_{Out}(t)$ are fed back to the input.

The magnetized sample can also be represented in terms of a second order system which comprises of an inductor *L* denoting the magnetic energy storage, a capacitor *C* denoting electrical energy storage and a resistor *R* representing the dissipative element. These variables related to an electrical circuit can be associated with a physical sample in magnetic resonance spectroscopy, where the time varying field generates a voltage which induces a current, a finite value of charge separation and dissipation. The physical effect of acoustic-Brownian interaction, which generates a voltage can be denoted by voltage $V_{in}$ (Fig. 6a). Using Kirchoff’s votlage law, we can write the following equation^42^,

18 $$ L\frac{d^2q}{dt^2}+R\frac{dq}{dt}+\frac{q}{C_a}=V_{{\rm in}} $$

The current is expressed as, $I=V_{{\rm in}}/(R+sL+1/Cs)$, where, $s=j\omega$, $j=\sqrt{-1}$ and $\omega$ denotes the angular frequency of fluctuation associated with thermal interactions. The voltage drop in the capacitor is $V_{{\rm Out(t)}}=(V_{{\rm in}}/Cs)/(R+sL+1/Cs )$. As the resonance frequency is expressed as, $\omega _n=1/\sqrt{LC}$ and $\zeta =R/(2L)$ denotes the damping coefficient, we can write the output voltage drop as,

19 $$ V_{{\rm Out}} (s)=V_{{\rm in}}(s)\frac{\omega _n^2}{s^2+2s\omega _n \zeta +\omega _n^2} $$

The time domain expression of Eq. 19 is written as,

20 $$ \frac{d^2V_{{\rm Out}}(t)}{dt^2}+2\zeta \omega _n\frac{d^2V_{{\rm Out}}(t)}{dt}+\omega _n^2V_{{\rm Out}}(t)=V_{{\rm in}}(t)\omega _n^2 $$

Its graphical illustration is represented in Fig. 6b where, $V''_{{\rm Out}}(s)$ represents the second derivative of the output voltage, while its first derivative is denoted by $V'_{{\rm Out}}(s)$. The output signal associated with, $2\zeta \omega V'_{{\rm Out}}(s)$, which is the dissipative element along with and the voltage from the capacitor, $V_{{\rm Out}}(t)$ are transferred to the input side as feedback voltage. The input voltage at a given time $t=t_0$ under thermal fluctuation can be modeled as $V_{{\rm in}}(t)=\sqrt{R k_BT\Delta f}\delta (t-t_0)$ where, $\delta (t-t_0) =1$ for $t=t_0$ and 0 for other values. Its Laplace transform is expressed as, $V_{{\rm in}}(s)=\sqrt{Rk_BT\Delta f}$. The net response of the system to thermal noise is expressed as,

21 $$ V_{{\rm Out}}(s)=\frac{\omega _n^2}{(s^2+2s\omega _k \zeta +\omega ^2)} \sqrt{Rk_BT\Delta f} $$

The voltage represented by Eq. (21) denotes the total response of the system arising as a consequence of an interaction between the input voltage associated with thermal fluctuations with macroscopic physical parameters of the material which is expressed using its transfer function. It demonstrates the fact that selective modes are enhanced under thermal fluctuations leading to a sudden surge in noise.

The magnitude of voltage generated under Johnson noise at $T=300\,\hbox {K}$, a damping coefficient of $\zeta =0.07$ and a bandwidth $\delta f= 1 \,\hbox {MHz}$ is $0.64\,\upmu \hbox {V}$ assuming a load of 100 $\Omega$. It tends towards values in the range of $30 \,\upmu \hbox {V}$ around the resonant frequencies which is higher by a couple of orders of magnitude around the resonant modes. The speed of sound in human tissue $v_s$ can be safely assumed to be $1500\,\hbox {m/s}$^44^. For a slice of tissue of length $L=10\,\hbox {cm}$, the fundamental frequency mode is $f=v_s/(2L) =7.5\,\hbox {kHz}$. The frequency of higher modes are $15\,\hbox {kHz}$, $22.5\,\hbox {kHz}$ and $30\,\hbox {kHz}$.

**Figure 7:**
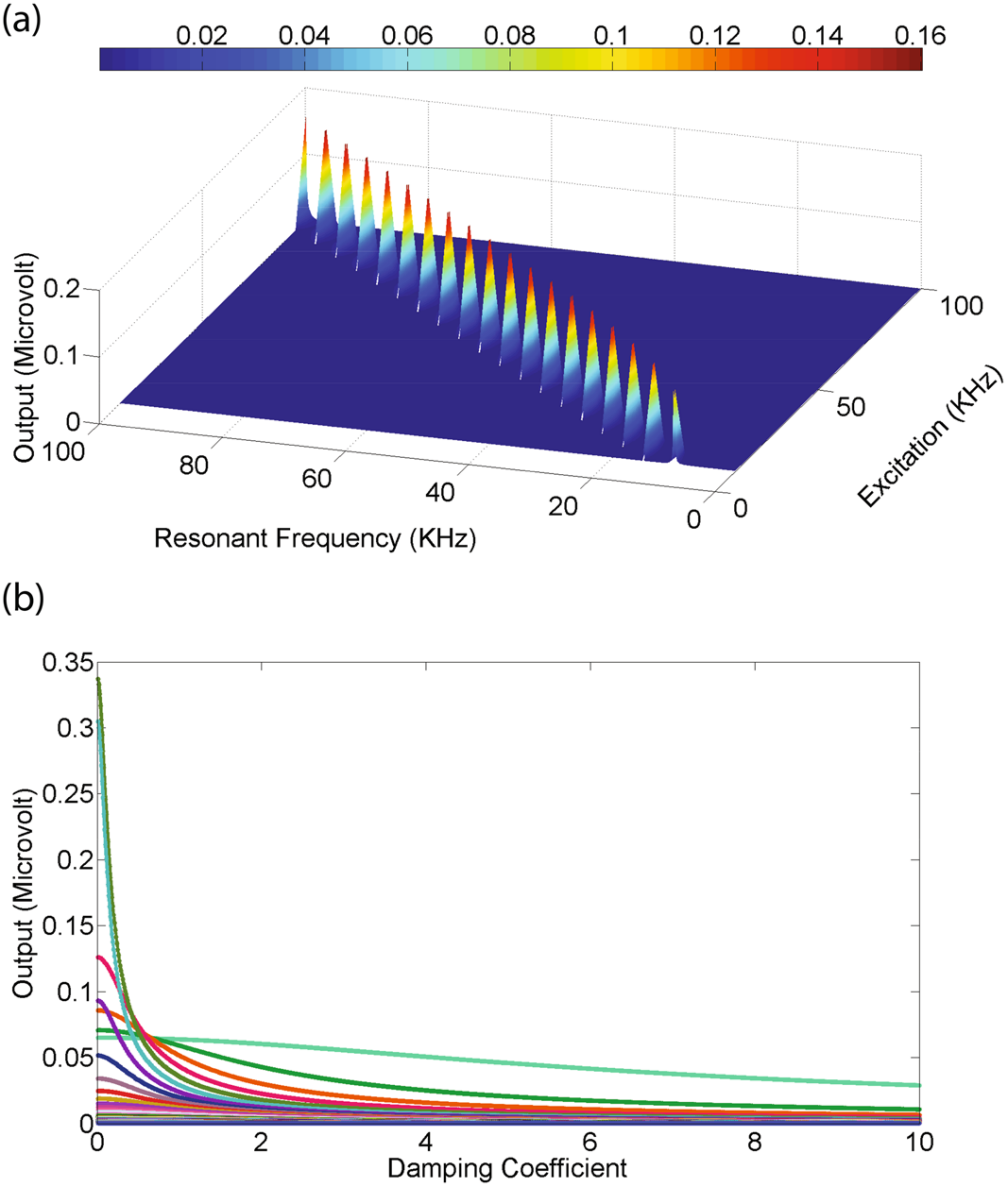
Energy enhancement under feedback. (**a**) Thermal fluctuations from a thermal source get coupled to magnetized sample leading to generation of noise which has a broadband frequency. However, under resonant excitation, the specific modes can get enhanced as shown in the graph which rise up to 0.16 $\upmu$V. The resonant modes are between 10 and $100\,\hbox {kHz}$ and the excitation frequency was varied from 100 Hz to $100\,\hbox {kHz}$. The spectral bandwidth of noise is $1\,\hbox {MHz}$ (**b**) The output signal at resonance decreases with an increase in damping coefficient from 0.01 to 10. The graph illustrates noise at a resonant frequency of $10\,\hbox {kHz}$ while the noise spectrum is varied from $1\,\hbox {kHz}$ to $1\,\hbox {MHz}$.

If a sample of human tissue of dimensions of the order of $10\,\hbox {cm}$ is subjected to a thermal fluctuations with a noise spectral frequency in the range of $100\,\hbox {Hz}$ to $100\,\hbox {kHz}$ and it has resonant modes between $10\,\hbox {kHz}$ to $100\,\hbox {kHz}$, the expected output signal is obtained by using Eq. 21. Its graphical illustration for these values is presented in Fig. 7a. It illustrates how resonant modes enhance the specific modes transforming the broadband noise generated under thermal fluctuations into narrow band signals.

According to Eq. 21, an increase in the value of damping coefficient, reduces the output signal which is graphically illustrated in Fig. 7b. The output voltage is calculated at thermal fluctuation frequencies ranging from $1\,\hbox {kHz}$ to $1\,\hbox {MHz}$ while the resonance frequency is assumed to be fixed at $10\,\hbox {kHz}$, while the damping coefficient is assumed to increase from 0.01 to 10. Thus, the Johnson noise which is characterized by a uniform spectral density and dependent on temperature, bandwidth and resistance assumes a new form and is defined by the resonant modes of the system.

**Figure 8:**
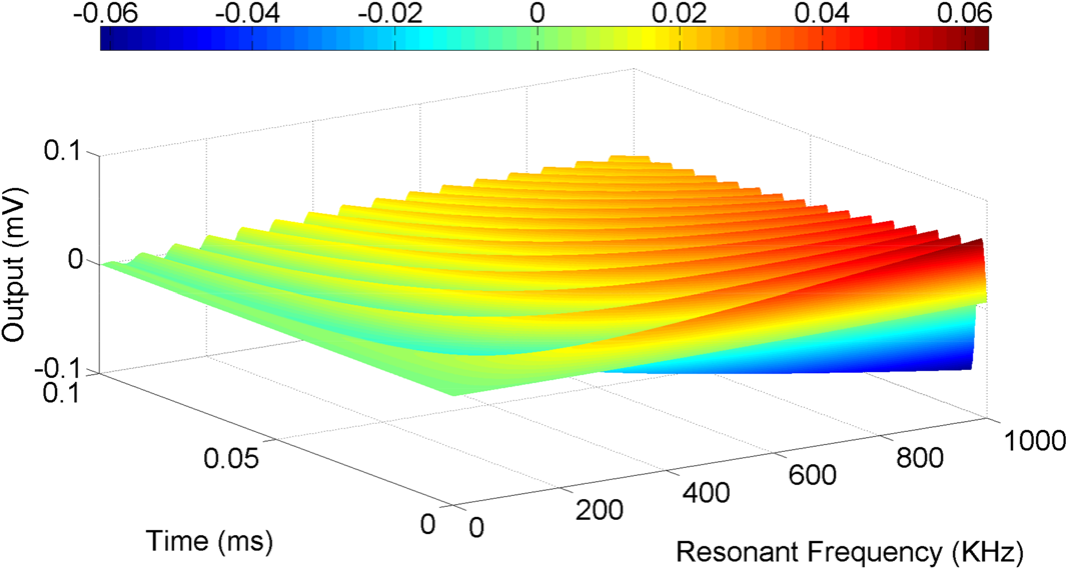
Time domain illustration of signal output under damping. The output voltage varies from $\pm 0.06$ mV to 0.

Its time domain solutions while assuming $0<\zeta <1$, are given by,

22 $$ V_{{\rm Out}}(t)=\frac{\omega _n}{\sqrt{1-\zeta ^2}}e^{-\zeta w_n t}\left[ { \sin (\omega _n\sqrt{1-\zeta ^2})t}\right] $$

A graphical illustration of the output voltage is shown in Fig. 8 where the resonant frequency is varied from $10 \,\hbox {kHz}$ to $1000 \,\hbox {kHz}$ and the time is varied from $0.1\,\upmu$s to 0.1 ms. For, $\zeta =1$, $V_{Out}= \omega ^2te^{-\omega _n t}$. For $0<\zeta <1$, the solution is,

23 $$ V_{{\rm Out}}(t)=\frac{\omega _n}{2\sqrt{\zeta ^2-1}}e^{-(\zeta -\sqrt{\zeta ^2-1}) w_n t} -\frac{\omega _n}{2\sqrt{\zeta ^2-1}}e^{-(\zeta +\sqrt{\zeta ^2-1}) w_n t} $$

For a general system consisting of a number of resonant frequencies, say $\omega _k$, where $k=1,\dots , N$, we can write,

24 $$ V_{{\rm Out}}(s)=\prod _{k=1}^{n}\frac{1}{(s-\omega _k)}V_{{\rm in}}(s). $$

This is a general expression which is valid for a multi-order system. An NMR system can be defined as a second order system having two resonant frequencies, hence the mathematical model presented here can be used to predict noise levels in the system.

## Comparison with Nyquist–Johnson noise

Noise generated in a dissipative system has a uniform spectral density over its frequency range^18,19^. The expression of Nyquist–Johnson noise is $V=\sqrt{4Rk_BT\Delta f}$ and its value over a bandwidth of 1 MHz, at a temperature $300\,\hbox {K}$ in a resistor of $50 \Omega$ is $0.90\,\upmu \hbox {V}$. It is comparable to acoustic noise under thermal fluctuations as illustrated in Fig. 7. However, an important issue is that, the noise is attenuated with time and is dependent on frequency, which can be selectively filtered out.

The nature of Nyquist–Johnson noise changes when there is a potential well associated with the particle. For a particle of mass $m_m$ placed in a potential well, the thermal fluctuations result in an increase in random vibration around the mean value. The Langevin equation can be written as^43^,

25 $$ \frac{d\varvec{v}}{dt}=-\frac{\alpha }{m_m}\varvec{v}-\omega ^2 \varvec{x}+\frac{\mathfrak {R}(t)}{m_m} $$

where $\varvec{v}$ is velocity, $\alpha$ is the drag coefficient, $\omega _0$is resonant frequency of the particle in a given potential well, $\varvec{\mathfrak {R}}(t)$ is the random fluctuating function representing interaction with the thermal source such that, $\langle \mathfrak {R}(t_1)\mathfrak {R}(t_2) \rangle =2\alpha KT \delta (t_1-t_2)$ . The Fourier transform of the Langevin equation is expressed as,

26 $$ \varvec{S}_{\mathfrak {R}(t)}=\int _{-\infty }^{\infty }dt e^{j\omega t}\langle \mathfrak {R}(t)\mathfrak {R}(0) \rangle =2\alpha k_{\mathrm {B}}T $$

Taking the Fourier transform of Eq. 26 and separating the terms, we can get the expression for displacement,

27 $$ \varvec{x}(\omega )=\frac{1}{m_m} \frac{\mathfrak {R}(t)}{|\omega _0^2-\omega ^2-j\alpha \omega /m_m|} $$

As the spectral density is proportional to $|x(\omega )|^2$, we can write,

28 $$ |x(\omega )|^2=\frac{1}{m_m^2} \frac{S_{\mathfrak {R}}(\omega )}{|\omega _0^2-\omega ^2-j\alpha |}=\frac{1}{m_m^2} \frac{2\alpha k_BT}{|\omega _0^2-\omega ^2-j\alpha \omega /m_m|} $$

**Figure 9:**
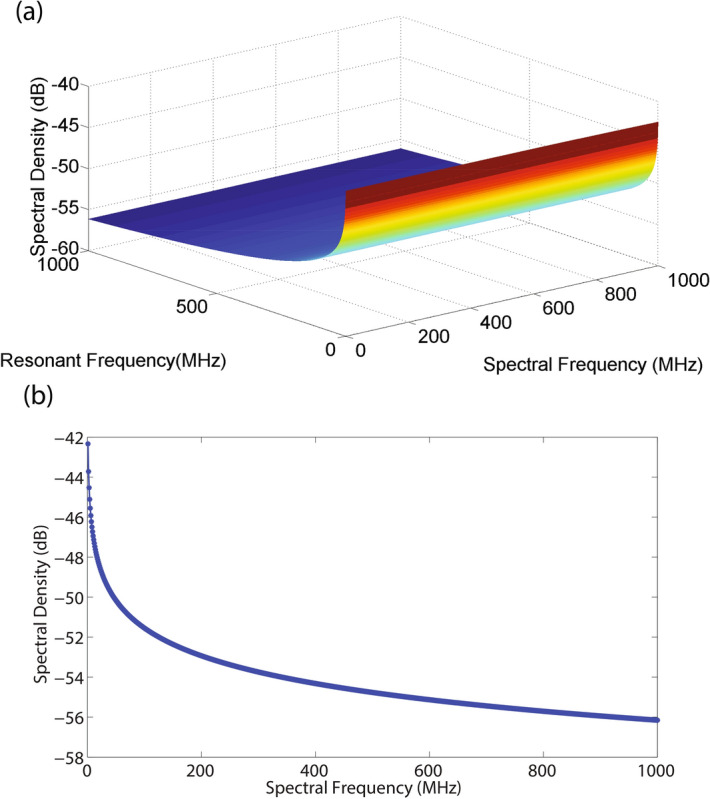
Brownian noise of a particle in a potential (**a**) Variation of spectral density with resonant frequency of the particle and spectral frequency. Noise has a uniform spectral distribution with frequency,however, there is an increase in noise with a reduction in resonant frequency of the system. Here, the spectral and and resonant frequencies are varied from 0 to 1 GHz. (**b**) Spectral density of noise reduces with an increase in time saturating to a final value steady state value. Here, the spectral frequency is varied from 0 to $1\,\hbox {GHz}$.

The spectral density plot on frequency and resonant frequency is found by using Eq. 28. It is illustrated in Fig. 9a where the amplitude decreases with an increase in frequency. The spectral as well as resonant frequencies are varied from 0 to 1 GHz.

The time domain dependence of spectral density is shown in Fig. 9b which illustrates a decaying form factor of noise where spectral frequency is varied from 0 to $1\,\hbox {GHz}$. A comparison of Fig. 4 and Fig. 9, indicates that the nature of acoustic-Brownian noise is similar to Brownian noise associated with a particle in a potential well.

## Discussion

A comparison between the values of magnetization under a static magnetic field of 1 T and magnetization induced by acoustic fields under thermal fluctuations indicates that the latter’s impact is directly dependent on the acoustic frequencies associated with mechanical modes. If the acoustic resonant modes are closer to the precession frequency under a given value of static magnetic field, the acoustic fields would play a dominant role. Thus, such effects can become subtle and strong in resolving tissue features whose dimensions are below $1\,\hbox {mm}$, where the acoustic modes are relatively higher. Another key issue is that such effects become dominant at short time scales, which implies that the Fourier components in the frequency domain may have a dominant impact. When considers the values of noise in terms of induced voltage, under feedback, the values are comparable to Nyquist Johnson noise. The analysis and the results on acoustic-Brownian magnetisation noise as a consequence of thermal fluctuations underscores the fact that special measures need to be integrated with NMR systems in order to reduce the effect of low frequency noise in order to improve signal to noise ratio. The noise levels in an NMR system is dominant not only at low frequencies but also in the nanosecond regime which can have a dominant impact on the overall signal to noise ratio. As the signal level is relatively high in the initial phase of nuclear relaxation^45^, the fact that this phase is also associated with high noise needs to be incorporated in noise models in NMR. Hence, there is a need to incorporate filter circuits addressing low frequency noise associated with the magnetization of a sample in NMR spectroscopy which can result in a better signal to noise ratio.

The other aspect of the current analysis on acoustic noise underscores the importance of noise enhancement under feedback which is a function of the existing resonant modes of the system. In the context of human tissues, the speed of sound is in the range of $1500\,\hbox {m/s}$ and the fundamental resonant mode in a section of tissue having a length of $1\,\hbox {cm}$ would be of the order of $1.5 \,\hbox {MHz}$ which may not hold much importance in the context of current MRI techniques which operate above 40 MHz. However, for diagnosis of sections of special components of tissues where the dimensions are in the range $1\,\hbox {mm}$, which is especially the case associated with early stage tumour growth^46^ and skin cancer^47^, the fundamental acoustic mode can be in the range of 15 MHz and higher modes at 30, 45 and 60 MHz can lead to noisy output under the context of MRI based diagnosis. The progression of tumour in tissues is associated with a change in mechanical stiffness^49–50^ which can change the nature of existing resonant modes^51^.

## Conclusion

Thermodynamic fluctuations get coupled to the magnetic moment of magnetized atoms, ions or molecules through shear stresses generated as a consequence of generation of acoustic waves resulting in acoustic-Brownian magnetization noise. Its physical nature has a spectral dependence which is similar to the Brownian noise associated with a particle in a potential well. The noise is dominant at small frequencies, lower dimensions and in the nanosecond regime before saturating to a steady value. There is enhancement of noise around the resonant frequencies of the system which offers a new dimension towards development of algorithms on noise filtering in NMR systems.
